# Supplementary material for: MetaRibo-Seq measures translation in microbiomes
Source: Nat Commun. 2020 Jun 29;11:3268. doi: 10.1038/s41467-020-17081-z (PMC7324362; doi:10.1038/s41467-020-17081-z)
Supplement: Supplementary file 10 — Supplementary Data 7 [file 41467_2020_17081_MOESM10_ESM.zip › File2/Confidence_High_Taxonomy/92396_out.krona.html]

Javascript must be enabled to view this page.

members
magnitude
magnitudeUnassigned
count
unassigned
taxon
rank

92396\_out

11

4

SRS017215\_contig\_number\_contig-100\_13090.40321SRS024318\_contig\_number\_contig-100\_23336.23336.23336SRS078425\_contig\_number\_contig-100\_8662.8662SRS893333\_contig\_number\_contig-100\_17458.17459

2
7
superkingdom

phylum
976
7

7
200643
class

order
171549
7

171552
3
family


SRS143214\_contig\_number\_1221
genus
1
838


SRS013946\_contig\_number\_2839SRS097867\_contig\_number\_841
species
2
1852368

family
815
2

816
2
genus

1347393
2
species

SRS023964\_contig\_number\_7226SRS1055060\_contig\_number\_contig-100\_131.457053

171551
2
family

2
836
genus

species

SRS893334\_contig\_number\_4779
41976
1

1
997829

SRS019329\_contig\_number\_247
species
